# Improvements in pig agriculture through gene editing

**DOI:** 10.1186/s43170-022-00111-9

**Published:** 2022-06-21

**Authors:** Kristin M. Whitworth, Jonathan A. Green, Bethany K. Redel, Rodney D. Geisert, Kiho Lee, Bhanu P. Telugu, Kevin D. Wells, Randall S. Prather

**Affiliations:** 1 Division of Animal Science, College of Agriculture Food and Natural Resources, University of Missouri, 920 East Campus Drive, Columbia, MO 65211 USA; 2 United States Department of Agriculture – Agriculture Research Service, Plant Genetics Research Unit, Columbia, MO 65211 USA

**Keywords:** Gene editing, CRISPR/Cas, Swine, Agriculture

## Abstract

Genetic modification of animals via selective breeding is the basis for modern agriculture. The current breeding paradigm however has limitations, chief among them is the requirement for the beneficial trait to exist within the population. Desirable alleles in geographically isolated breeds, or breeds selected for a different conformation and commercial application, and more importantly animals from different genera or species cannot be introgressed into the population via selective breeding. Additionally, linkage disequilibrium results in low heritability and necessitates breeding over successive generations to fix a beneficial trait within a population. Given the need to sustainably improve animal production to feed an anticipated 9 billion global population by 2030 against a backdrop of infectious diseases and a looming threat from climate change, there is a pressing need for responsive, precise, and agile breeding strategies. The availability of genome editing tools that allow for the introduction of precise genetic modification at a single nucleotide resolution, while also facilitating large transgene integration in the target population, offers a solution. Concordant with the developments in genomic sequencing approaches, progress among germline editing efforts is expected to reach feverish pace. The current manuscript reviews past and current developments in germline engineering in pigs, and the many advantages they confer for advancing animal agriculture.

## Background

The objective of this review is to highlight the improvements in swine welfare and agricultural efficiency by using gene editing technology.

## Introduction

Genetic improvement in pigs has traditionally been achieved via selective breeding of genetic outlier animals. Selective breeding has been very successful, and over the past 50 years has resulted in significant improvements in both the composition and efficiency of pig production. Continued genetic improvements can be made as long as beneficial natural genetic variation exists within the population. However, where there is no genetic variation, there is no room for selection and improvement of traits. Novel genetic variation and exogenous traits from outside of the genera and species can now be introduced into pig and other livestock species by using gene editing technologies. Types of variation may include altering a sequence to change the level of expression (from high to low or even off, i.e., a knockout), or to introduce genetic variation from another species. The technology to create such modifications via homologous recombination (e.g. meganucleases, zinc-finger nucleases, and Tal-effector nucleases) have been in the literature for the last 20–30 years. However, each of these technologies are cumbersome and inefficient. During the last decade, the Clustered Regularly Interspaced Short Palindromic Repeat (CRISPR)/Cas9 (CRISPR- associated nuclease 9) or CRISPR/Cas9 system has been repurposed to provide a highly efficient and relatively easy to use method for introducing targeted genetic variation in pigs.

In archaea and bacteria, CRISPRs function as a defense mechanism against bacteriophages via interactions with the Cas enzyme family. The Cas proteins create double stranded breaks in the genome of foreign DNA thus destroying the genome of the invading bacteriophage (Barrangou et al. 2007; Wiedenheft et al. 2012). This system is guided via sequence homology to genome sequences adjacent to protospacer motifs (PAMs). Several years after the initial discovery, this precise cleavage system was repurposed for genetic engineering, and the CRISPR revolution has since exploded (Cong et al. 2013; Cong and Zhang 2015). It did not take long for livestock genetic engineers to use this technology to design and create livestock with specific DNA edits that could improve animal agriculture. In 2014, cluster of differentiation (*CD163*) null pigs were created by using the CRISPR/Cas9 system (Whitworth et al. 2014). These CD163 knock out pigs were fully resistant to the virus that causes porcine respiratory and reproductive syndrome (PRRS) (Whitworth et al. 2016). In addition to creating pigs with disease resistance traits, gene editing can be used to improve production traits (Telugu et al. 2017), to lessen the impact on the environment (Forsberg et al. 2013), improve digestion (Guan et al. 2017) as well as to understand reproductive performance (Johns et al. 2021) Pigs are also used as models for evaluating somatic cell genome editing, as well as for gene therapy (Korpela et al. 2021; Liu et al. 2021). Gene editing is explored across the livestock industry to improve animal welfare and increasing efficiency. The objective of this review is to highlight these improvements that are specific to swine.

## Genome editing tools

The development and application of genome editing technology has rapidly improved the efficiency of genetically modified pig production. In the past 15–20 years, many different genome editors have been developed. This section will briefly describe some of the technologies that have revolutionized the speed and accuracy of creating genetic improvements. These genome editing technologies will only be briefly discussed here as there are many exhaustive reviews, which can be found elsewhere (Lee et al. 2019, 2020; Yang and Wu 2018; Sander and Joung 2014; Redel and Prather 2016; Ryu et al. 2018). The basis of genome editing is its ability to rely on the use of nucleases that are directed to a specific location in the genome, which then cleave DNA and create double-strand breaks (DSBs). These DSBs are then repaired by the cell’s natural repair mechanisms. The highly efficient and predominantly used cellular pathway to repair DSBs is the non-homologous end joining (NHEJ) pathway. NHEJ is error prone leading to the production of small insertions or deletions (indels). The indels created by NHEJ can vary in length and may cause frameshift mutations in the coding sequence or deletion of translational start sites both of which lead to gene knock outs. When multiple locations in a gene are targeted by the CRISPR/Cas9 system simultaneously, the CRISPRs can result in deletion or inversion of the intervening sequences, similarly resulting in gene knock outs. If donor template or oligonucleotides are present, the cell can utilize the homology-directed repair (HDR) pathway to introduce site specific modifications from the donor template at the nucleotide level. This can allow for insertion of a transgene, swapping of exons, or even single nucleotide changes in the cell of interest (Whitworth et al. 2014; Wells and Prather 2017). However, the efficiency of introducing intended modifications in cells or embryos by HDR is often very low.

Zinc finger nucleases (ZFNs) were one of the first of these technologies to provide a systematic approach to target nearly any sequence and to be adapted for genome editing in mammalian cells (Urnov et al. 2005). This genome editing tool is comprised of two different domains, a zinc finger DNA binding domain and a *Fok1* nuclease cleavage domain (Song et al. 2014). For ZFNs to be able to cleave DNA, the *Fok1* nuclease must dimerize to become active. Therefore a pair of zinc fingers are required to align over the specific location in the genome to be cut (Vanamee et al. 2001). The zinc finger DNA binding domain allows the recognition of specific locations in the genome, as each zinc finger recognizes a specific three nucleotide sequence. Generally, zinc fingers are created in a series of 3–15 repeats, where the number of DNA binding motifs directly affects the sequence specificity (Rutherford et al. 2013). Transcription activator-like effector nucleases (TALENs) were the next genome editing tool developed and contain an improved specificity of editing in mammalian cells. Like ZFNs, TALENs contain both a DNA binding domain and a *Fok1* nuclease domain. However, the DNA binding domain allows TALENs to be more specific as this domain consists of multiple tandem repeats. Each repeat comprises 33 to 35 conserved amino acids with a specific di-residue conferring specificity for binding to a base pair (Deng et al. 2012).

While the development of these endonucleases improved the production of genetically modified livestock, it was the discovery and optimization of the CRISPR/Cas9 system that revolutionized this process. The CRISPR/Cas9 system is adapted from *Streptococcus pyogenes* in which it was used as a defense system to protect against invading plasmids and viruses (Wiedenheft et al. 2012; Rodríguez-Rodríguez et al. 2019; Jinek et al. 2012). The ability of the bacterial cell to utilize this defense system to induce DSBs at a specific location was then adapted to be used as a RNA-guided genome editing system (Ryu et al. 2018). The CRISPR/Cas9 system is comprised of a single guide RNA, formed from a combination of CRISPR RNA and the transactivating RNA that is homologous to a targeted region of a chromosome. The guide RNA directs Cas9 nuclease to a target site which then cleaves the DNA if a protospacer adjacent motif (PAM) sequence is present (Cong et al. 2013).

Although the CRISPR/Cas9 system has revolutionized the ability to make genome edits, it has limitations in its use to insert a specific DNA sequence into the genome. The ability to insert a specific DNA template by using the cell’s natural HDR mechanism has low efficiency of insertions and high rates of indels (Mao et al. 2008a). More recently, an advancement in genome editing which utilizes some of the CRISPR components with modified enzymes to directly cause point mutations into cellular DNA or RNA without creating DSBs has been developed to circumvent this limitation (Gaudelli et al. 2017; Komor et al. 2016, 2017). Two different classes of base editors (BE) have been developed: cytosine base editors and adenine base editors. The first-generation base editor developed, BE1, contains a catalytically inactive dead Cas9 (dCas9) which is conjugated to a cytosine deaminase. This BE1 can then deaminate cytosine to uracil and the cell replication machinery then recognizes the uracil as a thymine, resulting in a C-to-T or a G-to-A substitution, depending on which DNA strand is targeted (Kantor et al. 2020). A second generation base editor, BE2, was then developed to increase the efficiency of U.G pair to a T.G pair by fusing a uracil DNA glycosylase inhibitor to the *C*-terminus of BE1 (Rees and Liu 2018). The third generation of base editors, BE3, uses a Cas9 nickase (nCas9) instead of a dCas9 to make point mutations. This nCas9 “nicks” the DNA and utilizes the cells mismatch repair pathway to produce the edit (Marx 2018). A fourth-generation editor, BE4, was created by co-expressing BE3 with uracil DNA glycosylase inhibitor to increase the programmable C.G to T.A. base pair conversion not seen by BE3 (2017). While the use of base editing can efficiently produce four transition mutations: C-to-T, G-to-A, A-to-G, and T-to-C, the eight transversion mutations are not permitted (Kantor et al. 2020). Other adaptations to these editors have also been made and are currently being studied.

To avoid the limitations of base editing, a new technology called prime editing, was developed and mediates insertions, deletions, and all 12 possible base-to-base conversions without requiring DSBs or donor DNA templates (Anzalone et al. 2019). This approach minimizes the off-target effects that are often seen in normal CRISPR/Cas9 editing and also improves the target specificity in genomes (Anzalone et al. 2019). Prime editing utilizes a Cas9 nickase (nCas9) which contains one inactive DNA cleavage domain that is fused with a reverse transcriptase (RT-nCas9), which can be transfected along with a prime editing guide RNA (pegRNA). One of the important differences in this system compared to the conventional CRISPR/Cas9 system is that the pegRNA not only identifies the complimentary sequence of the target, like single guide RNA (sgRNAs), but it also contains an additional sequence that will replace the target DNA nucleotides (Anzalone et al. 2019). Upon target recognition, the PAM containing strand is nicked and the pegRNA binds to the nicked strand (Geurts et al. 2021). A DNA flap with a 3’OH group is produced and serves as a primer for the RT, which extends the 3’ flap by copying the edit specific sequence of the pegRNA (Scholefield and Harrison 2021). Prime editors have been successfully used in human HEK293T cells with ranges of 20–50% editing efficiency (Anzalone et al. 2019) and in mouse cells (8–40% efficiency) and zygotes (44–75% efficiency) (Liu et al. 2020). This is just one more advance in the CRISPR/Cas9 technology that is currently being studied; it has yet to be used as a method to produce genetically modified livestock.

## Germline editing

Germline editing is “the process by which the genome of an individual is edited in such a way that the change is heritable” (Sorrell and Kolb 2005). As the name suggests, for the genetic modification to be heritable, the germline of the individual will need to be modified as well. There are two major means for germline engineering: (1) embryo-based and (2) cell-based approaches. Each of these have their inherent advantages and disadvantages and are briefly discussed below.

### Embryo-based germline editing

In a landmark publication, Gordon et al. reported for the first time the generation of a transgenic mouse, ushering in a new era of germline engineering in mammals (Gordon and Ruddle 1981). The procedure is simple and elegant and requires the assembly of a DNA construct containing the genes of interest under the control of appropriate regulatory elements, microinjection of the DNA construct into the pronucleus of the donor zygotes, and implantation of microinjected zygotes into pseudo-pregnant recipient animals for the delivery of transgenic founder animals. As expected, the outcomes of zygotic injections are often random, and range from no integration to integration of multiple copies into the genome, a lack of control over the number of integrated transgenes and consequently the level of transgene expression, high degree of mosaicism, and insertional mutagenesis to name a few. The approach thereby necessitates generating multiple transgenic founder animals to screen for optimal transgene copy number integration, expression, and transmission to the next generation. The application of this approach across species is not straightforward. For example, the use of this method in pigs is limited due to the high lipid content in the zygotes, which obscures the pronuclei (Perry et al. 1999; Salamone et al. 2018) for transgene injection. Notwithstanding the drawbacks, zygotic injections remained the only option for genetic manipulation for two decades and has been employed with varying success in pigs (Hammer et al. 1985; Hofmann et al. 2003; Whitelaw et al. 2004).

The availability of CRISPR/Cas and other programmable editors has provided much needed stimulus for germline engineering/editing efforts in pigs. For loss-of-function studies, gene knock out animals can be generated by microinjection of the editors directly into the oocytes (Su et al. 2019) or zygotes (Hai et al. 2014). The ready availability of relatively inexpensive and off-the-shelf CRISPR reagents, alongside the ease of delivery of the reagents (microinjection or electroporation) has democratized germline editing efforts in pigs and other livestock species across the globe. Conceptually, a reproductive biologist well versed in the procedures of in vitro fertilization or harvesting in vivo fertilized embryos and embryo transfers can perform germline editing. Added to this, the i-GONAD approaches (Takabayashi et al. 2018), which lend to the delivery of reagents into the oviduct; electroporating zygotes further lowered the barrier for germline editing (Takabayashi et al. 2018). Another greater advantage of editing in zygotes is the ease of access of the editors to the target gene of interest, especially those residing in the heterochromatin state in somatic cells. This is in part due to the unwinding of the maternal and paternal chromosomes and the relatively relaxed state of chromatin prior to pronuclei formation. When the zygotes are recovered from in vivo fertilized zygotes, the efficiencies of pregnancies are quite high, with the added benefit of introducing intended genetic modifications directly in the desired genetics. Recent developments in in vitro culture and fertilization of porcine zygotes with efficiencies reaching closer to in vivo derived embryos is expected to expand our germline editing efforts (Yuan et al. 2017). Nevertheless, barring the ease of delivery, a major limitation of this approach is the underlying mosaicism, with the resulting offspring bearing distinct somatic vs germline modifications. This makes the task of screening heritable mutations in a large animal model like pigs, tricky. The incidence of mosaicism is especially more pronounced in methods involving HDR-based targeting (Park et al. 2019, 2020). Several approaches have been proposed or being tested, which include (1) modifying reagent concentrations, (2) choice of reagents, (3) modification of targeting oligos/plasmids, (4) altered injection times, and (5) use of CRISPR inhibitors, to name a few. However, it is to be noted that every allele and trait needs independent validation, and it is prudent to perform diligent in vitro validation assays prior to committing to embryo transfers and the associated long time for gestation and reaching puberty.

### Cell-mediated approaches

1. Embryonic stem cell (ESC)-based editing
  The discovery of ESC represents another landmark event in germline engineering efforts. The ESC are derivatives of inner cell mass-a cluster of pluripotent cells in the early blastocyst that gives rise to the fetus proper (Evans and Kaufman 1981). In rodents, the ESC can be reliably and stably established in culture; permit multiple and complex genetic modification(s); propagated and selected over multiple generations. When injected into donor blastocysts, the ESC contribute to multiple lineages including germ cells in the resulting chimeric offspring. The discovery of ESC in mice has been a game changer for germline engineering efforts, and catapulted the mouse as a prime genetic model. Much of this excitement and promise, however, did not translate to pigs due to a lack in the availability of authenticated and chimera competent stem cells (Malaver-Ortega et al. 2012; Park and Telugu 2013). Induced pluripotent stem (iPS) cells with characteristics/potency similar to that of ESC (Takahashi and Yamanaka 2006), and a theoretical possibility of generating these lines from multiple species offered hope. In fact, iPSC derived germline competent offspring were produced in mouse and rat (Hamanaka et al. 2011; Okita et al. 2007). However, attempts to replicate these findings in pigs were proven to be less successful with only one reported study in pigs (West et al. 2010) with no follow-up. Likewise, they have proven to be ineffective as nuclear donors for somatic cell nuclear transfer (SCNT) (Fan et al. 2013). The lack of availability of robust stem cells alongside relatively long generation intervals compared to rodents, has resulted in limited or no adoption of this erstwhile promising approach for pig germline editing.
2. Somatic cell nuclear transfer (SCNT)
  The history of nuclear transfer predates zygotic injections. Hans Spemann first theorized SCNT in 1938. Briggs and King successfully demonstrated nuclear cloning by transplanting a nucleus from a blastula stage frog embryo into the cytoplasm of an enucleated frog egg, in 1952 (Briggs and King 1952). Illmensee in 1981, generated clonal mice from SCNT, however, this claim has not been validated following further investigation (Illmensee and Hoppe 1981). Fifteen years after the generation of the first transgenic mice, and almost 30 years after cloning an amphibian, Campbell et al., succeeded in generating a cloned mammal- a sheep named dolly (Campbell et al. 1996). This was soon followed by success in pigs (Onishi et al. 2000; Polejaeva et al. 2000). SCNT involves several key steps, each of them having a potentially significant impact on cloning efficiency. These include: (a) genetic modification of donor cells; (b) removal of metaphase chromosomes from a MII-arrested oocyte (enucleation) by either aspiration or bisection; (c) transfer of donor cell nuclei, in which a donor cell is placed next to an enucleated oocyte and fused by an electrical pulse, or injected directly into the cytoplasm of the enucleated oocyte; (d) activation of the reconstructed embryos; and (e) embryo culture and transfer into synchronized surrogate recipients (Lai and Prather 2003, 2004) The procedure is technically challenging and expensive, making it less amenable for adoption by practitioners in the field. At a cellular level, the somatic cells typically chosen are fetal fibroblasts, which proliferate slowly and have a finite lifespan in the culture, low recombination rates ranging from 1 in 10^6^–10^7^ in cultured cells, and with hemizygosity often being the outcome (Park et al. 2019; Sedivy and Dutriaux 1999). Given the relatively long gestation periods (~ 4 months) and time to reproductive age in pigs (6–9 months), breeding of hemizygous founders to homozygosity makes it a cost prohibitive option Thus, often a round of embryo transfer is performed for harvesting the fetal fibroblasts with hemizygous modification followed by a second round of gene targeting and SCNT for generating offspring with biallelic modification. Technical challenges aside, SCNT efficiencies are typically low and often suffer from challenges emanating from incomplete reprogramming of the embryo and abnormalities in the resulting cloned animals following birth, which manifest as lameness, respiratory defects, immunodeficiency, obesity, and early death (Loi et al. 2016; Ogura et al. 2013). Although gene targeting efficiencies are lower in somatic cells, the introduction of a double stranded break at the target site, greatly improves the likelihood of gene targeting at the target site (1:1000 vs 1:10^6^–10^7^) (Rouet et al. 1994). These improved efficiencies ensure that complex and targeted genetic modification in homozygosity can be achieved from one round of gene targeting in vitro. When the pre-screened clonal lines are used for SCNT, the resulting litter of cloned animals will all carry the pre-ordained genetic modification. This, however, does not eliminate the inherent problems such as incomplete reprogramming in the resulting embryos. In the absence of credible alternatives, SCNT remained a method of choice for germline engineering in pigs.

In summary, embryo-based germline editing is preferred in the agricultural context, where editing can be performed in the elite founders in the nucleus herd, so that the resulting founders can be introduced into the breeding pipeline. Cell-based approaches are most used when the genetic modification needs to be introduced into rare genetics or less prolific breeds (Yucatan, Ossabaw, other minipigs), and when the goal is to qualify the phenotype resulting from loss-of-function or over-expression studies prior to deployment in a commercial setting. While there is a tradeoff with each approach, it is clear that the advent of genome editors has fueled adoption and deployment of germline engineering for addressing critical agricultural priorities. The types of genetic modifications that can be made are limited only by our imagination and biology. If one can imagine the modification, and the modification is compatible with biology, then it can probably be made. The increase in the functionality and utility of tools to make these genetic modifications are described in the next section.

## Editing pigs to confer disease resistance

Creating pigs that are resistant to devastating pig diseases has been successfully performed over the past 5 years. The first disease resistant model to be discussed is the *CD163* null pig which is fully resistant to both Type 1 (European) and Type 2 (North American) porcine reproductive and respiratory syndrome virus (PRRSVs) (Whitworth et al. 2016; Wells et al. 2017). As stated above, other disease resistant models have also been created including amino peptidase N (*ANPEP*) edited pigs that are fully resistant to the coronavirus transmissible gastroenteritis virus (TGEV), anthrax toxin receptor 1 (*ATNXR1*) edited pigs that are resistant to Senecavirus A (SVA) (Chen et al. 2022), and porcine radical SAM domain-containing 2 (*pRSAD2*) knock in pigs that are resistant to Classical Swine Fever Virus (Xie et al. 2020). An altered RELA proto-oncogene, NF-kB subunit (*RELA*) model was created to confer resistance to African Swine Fever Virus (McCleary et al. 2020). Another model, transmembrane serine protease (*TMPRSS2*) null was created to be resistant to a wide variety of viruses that include influenza (Whitworth et al. 2017). The details of those models will be reviewed below.

### PPRS virus resistant pigs

Porcine Reproductive and Respiratory Syndrome is caused by a positive-stranded RNA Arterivirus that is categorized by genotype as either Type 1 or Type 2 (Brar et al. 2015; Stadejek et al. 2013). Both now appear to have world-wide distribution and there is a highly pathogenic Type 2 PRRSV (hpPRRSV) prevalent in Asia (Shi et al. 2010). The Type 1 and Type 2 viruses are genetically distinct but appear to use the same entry mediator. PRRSV replicates in pulmonary alveolar macrophages (PAMs) and results in prolonged viremia, respiratory distress, lethargy, and a mortality rate of 12–15% in young pigs. There is severe reproductive failure in sows including a high rate of late term abortion, early farrowing, decreased litter size and increased number of mummies. In boars there is a reduction of libido, fever and lower sperm count (Schulze et al. 2013).

Determining the entry mediator was in high demand due the huge economic and animal well-being losses caused by PRRSV. One model for viral entry proposed by Van Breedam (Breedam et al. 2010) involved PRRSV having a loose association with heparan sulfate. As proposed, the virus then binds to the N-terminal region of sialic acid binding Ig like lectin 1 (SIGLEC1) followed by internalization into clathrin-coated vesicles followed by fusion to form endosomes. The pH in the endosome drops and then CD163 associates with the virus to uncoat the viral genome such that the genome is released into the cytoplasm of the PAM, resulting in infectivity (Gorp et al. 2008, 2010). Interestingly, Calvert et al., provided in vitro data that suggested that CD163 was the sole entry mediator for the PRRSV (Calvert et al. 2007).

To address the question of SIGLEC1’s role in PRRSV infection, *SIGLEC1* was knocked out in pigs and these animals were challenged with Type 2 PRRSV (Prather et al. 2013). It is interesting to note that creating the founder animals that were bred to produce the *SIGLEC1* null animals for the challenge was done prior to the availability of gene editors and took many years to accomplish. Macrophages from *SIGLEC1* null pigs retained the ability to express CD163 protein at the same level as heterozygous and wild-type littermates. Infection of *SIGLEC1* null pigs with Type II PRRSV resulted in a productive infection as demonstrated by the presence of both circulating virus nucleic acid and viable virus. The peak and duration of infection in *SIGLEC1* null pigs were no different from *SIGLEC1* heterozygous or wild-type littermates (Prather et al. 2013). While a null of *SIGLEC1* did not prevent infection, the SIGLEC1 protein may be redundantly involved in viral attachment to the PAM.

The second candidate entry mediator was CD163. CD163 is a member of the scavenger receptor cysteine-rich (SRCR) superfamily. The protein has 9 extracellular SRCR domains, a transmembrane domain and a cytoplasmic tail. There are 17 exons in *CD163*. Exon 7 encodes SRCR domain 5 of the protein. SRCR domain 5 appears to be the domain responsible for unpackaging of the genome of Type 1 viruses as deletion of other domains did not inhibit infection in vitro (Gorp et al. 2010). Replacement of domain 5 of CD163 with domain 8 of human CD163L also abolished Type 1 infectivity in vitro. The first PRRS resistant pigs were created by introducing indels into exon 7 of *CD163* that resulted in complete loss of function of the protein. When *CD163*^*−/−*^ pigs were challenged with either a Type 1 or Type 2 PRRSV there was no evidence of infection as measured by fever, coughing, viremia, antibody response or lung pathology (Whitworth et al. 2016; Wells et al. 2016). Similarly, when PAMs from *CD163*^*−/−*^ pigs were challenged in vitro with nine distinct Type 1 or six distinct Type 2 isolates there was also no evidence of infection. A follow-up experiment also determined that a pregnant *CD163*^*−/−*^ sow could protect her *CD163* heterozygous/PRRSV susceptible fetuses after PRRSV inoculation of the sow (Prather et al. 2017). Of note; collaborators at Kansas State University had challenged over 3000 pigs representing different genotypes from across the United States and had not found a naturally occurring PRRS resistant pig (Dekkers et al. 2017).

Excitingly, other groups began to repeat the CD163 experiments all over the world with different background genetics and PRRSV isolates, and the results continued to be consistent, with full resistance to PRRSV. Roslin Institute created pigs that removed exon 7 encoding the viral binding domain, but the remaining CD163 protein remained intact (Burkard et al. 2017, 2018). The pigs were fully resistant to both type 1 and type 2 PRRS. Another group challenged exon 7 deleted pigs with highly pathogenic type 2 PRRSV (hpPRRSV) common in China and also found the pigs to be fully resistant (Wang et al. 2019). Yang et al., created *CD163*^*−/−*^ pigs that were fully resistant to hpPRRSV (Yang et al. 2018). While these results are exciting and provide a solution to a problem that has no other solution, the gene edits still need to be approved by the appropriate government regulatory agencies before edited animals can enter the food chain. Described above is the bare minimum type of edit that can be made. An edit that removes something (CD163). No new protein is added. A few letters of a 3 billion letter genome are altered. A modification simpler than this is difficult to imagine, and in theory such modifications should be the easiest to gain regulatory approval.

### Coronavirus resistant pigs

Coronaviruses are enveloped, single-stranded, positive sense RNA viruses, placed in the order, *Nidovirales*. Coronaviruses have a unique structural feature called a corona that is formed by spike proteins protruding from the viral surface (Li et al. 2007). There are multiple coronaviruses that infect pigs that include the alphacoronaviruses TGEV and porcine epidemic diarrhea virus (PEDV). TGEV and PEDV infection of immunologically naïve newborn piglets results in losses approaching 100% mortality. Both viruses result in mal-absorptive diarrhea and dehydration caused by the destruction of infected gut enterocytes (Madson et al. 2016; Saif et al. 2012). In 2013, a PEDV outbreak in the United States resulted in the death of nearly 7 million pigs, an estimated 10% loss in U.S. pig production for that year (Stevenson et al. 2013). TGEV is typically less destructive in swine herds because a TGEV deletion mutant, porcine respiratory corona virus (PRCV) is endemic. Pigs typically recover well from PRCV infection and produce neutralizing antibodies that also neutralize TGEV. This cross reactivity results in a less severe infection in piglets exposed to TGEV (Kim et al. 2000). In 2014, another coronavirus deltacoronavirus (PDCoV) was isolated from five infected farms in Ohio and rapidly spread throughout the United States (Wang et al. 2014).

Porcine aminopeptidase N (APN, ANPEP, CD13) was characterized and hypothesized to be an entry mediator for pig coronaviruses. Porcine ANPEP is a 963 amino acid, type II membrane metallopeptidase responsible for removing N-terminal amino acids from protein substrates during digestion. A variety of cells and tissues have low levels of ANPEP expression, but it is highly expressed on enterocytes. Several studies have illustrated an interaction between spike protein of many coronaviruses and ANPEP (Ren et al. 2010; Kamau et al. 2017; Oh et al. 2003). Dot blot analysis also showed that both the N-terminal and C-terminal domain of TGEV and PEDV spike protein would hybridize to porcine ANPEP (Li et al. 2007). *ANPEP* null pigs were created at the University of Missouri with the CRISPR/Cas9 system (Whitworth et al. 2019). The resulting F1 offspring and wild type age matched counterparts were challenged with both PEDV and TGEV. *ANPEP* null pigs were fully resistant to TGEV infection but retained susceptibility to infection with PEDV. Immunohistochemistry confirmed the presence of PEDV reactivity and absence of TGEV reactivity in the enterocytes lining the ileum in *ANPEP* null pigs. *ANPEP* null pigs were also challenged with PDCoV both in vitro and in vivo (Stoian et al. 2020). This study showed that *ANPEP* null PAMs were fully resistant to PDCoV infection, but the lung fibroblast-like cells from the same pigs supported PDCoV infection at high levels. Similar to the fibroblast-like cells, the challenged ANPEP null pigs were susceptible to the PDCoV. This study highlights the importance of in vivo challenges when studying disease resistance. Double *CD163*/*ANPEP* null pigs were created by gene editing and challenged with both PRRSV and TGEV and were resistant to both while maintaining the same production level as wild type pigs (Xu et al. 2020). The double null pigs were also challenged with PDCoV and were not resistant similarly to the Stoian et al. experiments. There was a delay in onset of humoral immunity suggesting ANPEP may still be playing a role, but it is not solely responsible for infectivity.

### African swine fever resistance in pigs

African swine fever virus (ASFV) causes a lethal, hemorrhagic disease in domestic swine that threatens pig production across the globe. ASFV is a large, enveloped double-stranded DNA virus and the single member of the family *Asfarviridae*. Warthogs act as a host to the virus as it causes a non-clinical, persistent infection, i.e., they do not succumb to the otherwise highly lethal effects from infection as seen in other species. When the same virus infects domestic pigs that are used for food production, widespread systemic hemorrhage typically occurs followed by death within days (Blome et al. 2013). One of the differences between the warthog and domestic pigs is a three amino acid difference in the RELA protein, a subunit of the NF-KB transcription factor that plays a key role in regulating immune response upon infection. Gene edited domestic pigs were created with either 2 or 3 of these amino acid changes and were challenged with ASFV. There was no measurable difference in pigs with the 2 amino acid substitution, but pigs with all 3 warthog amino acids had a delayed onset of clinical signs and less viral DNA in blood and nasal samples (McCleary et al. 2020; Lillico et al. 2016). Other in vitro evidence indicated that the PRRSV receptor CD163 may be playing a role in ASFV infectivity. ASFV infected macrophages had an enhanced expression of CD163 and anti-CD163 antibodies could block infection of ASFV in macrophages in a dose dependent manner (Sanchez-Torres et al. 2003). In vivo challenge of *CD163* null pigs with the Georgia 2007/1 isolate of ASFV resulted in robust infection of the pigs ruling out a significant role for CD163 in infection (Popescu et al. 2017). To date, an on/off type of entry mediation as observed with PRRSV or TGEV resistance has not been identified for ASFV.

### Senecavirus A resistant pigs

Senecavirus A (SVA) is a non-enveloped, positive-sense, single-stranded RNA virus belonging to the genus Senecavirus in the family *Picornaviridae*. Clinical signs include vesicular lesions on the snout and coronary bands and increased mortality in newborn pigs. SVA was first reported in the United States in 2010 and by 2015 there were over 230 cases causing increased concern (Joshi et al. 2016). The clinical signs are the same as foot and mouth disease virus (FMDV) and swine vesicular disease virus (SVDV) which can be startling for producers until diagnostics can confirm the presence of SVA. Anthrax toxin receptor 1 (ANTXR1), also known as tumor endothelial marker 8 (TEM8) was identified to be the cellular receptor for SVA by using genome-wide loss of function screens (Miles et al. 2017). The same group demonstrated that ANTXR1 is necessary for permissivity after infection of cell lines with SVA. These clinical signs (vesicular lesions) also cause disruptions in animal flow as the symptoms are notifiable and testing must be done to rule out FMDV prior to animals leaving the facility.

Gene edited *ANTXR1* null pigs were created to investigate the role of ANTXR1 in SVA infection both by both in vitro and in vivo challenges (Chen et al. 2022). Fibroblast cell lines derived from *ANTXR1* null pigs and WT pigs were challenged with SVA and infectivity was supported only in the WT lines. An in vivo challenge was then performed and showed that clinical symptoms of SVA and circulating viremia were present in the infected WT pigs but were absent in KO pigs. The study challenged pigs with other genotypes where part of the protein was intact, and it was determined that if the N-terminus of ANTXR1 is kept intact, SVA infectivity will be reduced, but not diminished. When the entire ANTXR1 is removed the pigs do not get infected with SVA.

### Updates on other potential disease resistant models

Other models have been created to study disease resistance and receptor binding ability. One such model is a knock out of the transmembrane protease, serine S1, member 2 (*TMPRSS2*) (Whitworth et al. 2017). This model was created to address the role of TMPRSS2 protease in swine influenza pathogenesis. Influenza hemagglutinin is cleaved by host proteases, an essential step for infection (Hatesuer et al. 2013; Tarnow et al. 2014). It is thought that removal of this protease may prevent influenza infectivity. To date, there are no published reports of this model being challenged so it is unknown if the goal was achieved. There has also been progress in reducing infectivity of classic swine fever virus (CSFV) and pseudorabies virus (PRV), which are other economically important pig pathogens. Pigs were created with a site-specific knock-in of Radical S-Adenosyl Methionine Domain Containing 2 (*RSAD2*) gene by using CRISPR/Cas9. RSAD2, is a member of the radical S-adenosylmethionine superfamily of enzymes that has antiviral activity (Chin and Cresswell 2001). Both in vitro and in vivo challenges showed in cell lines and pigs over expressing pRSAD2 resulted in reduced CSFV and PRV infectivity (Xie et al. 2020). Another approach to reduce disease by gene editing is to create pigs that express a naturally occurring antimicrobial human lysozyme (hLZ) in the milk. Piglets that consume the milk recover more quickly from bacterial induced diarrhea (Lu et al. 2014; Huang et al. 2018). As our knowledge of viral entry mediators increases, the ability to design pigs to prevent infectivity will also increase. To show that multiple diseases can be prevented in single animals we have stacked three gene edits (*CD163*, *SIGLEC1* and *ANPEP*) conferring disease resistance in the same animals. Superficially, these animals appear normal, and we have observed no undesirable effects (Whitworth, unpublished results).

## Production traits

A few different genetically modified pigs have been produced to exhibit improved production traits. A few examples are listed below.

### Improved thermoregulation

Piglets lack thermoregulation due to a non-functional uncoupling protein 1 (*UCP1*) gene, which plays a role in thermoregulation and adiposity. A genetic event occurred about 20 million years ago that resulted in the loss of exons 3–5 of *UCP1* in pigs (Berg et al. 2006). In a commercial setting, sows are farrowed in crates supplemented with heat pads and heating lamps, which increases utility and production costs. To restore *UCP1* function, pigs were genetically modified by using CRISPR/Cas9 to knock-in mouse adiponectin-*UCP1* in the pig endogenous *UCP1* locus (Zheng et al. 2017). These pigs exhibited improved thermoregulation during acute cold exposure and decreased fat deposition, without altering physical activity or daily energy demands (Zheng et al. 2017).

### Changes to body composition of carcass and meat quality

Improvements to meat quality have been achieved by expressing a fatty acid desaturase gene from spinach to increase the levels of linoleic acid in pigs. Mammals lack the desaturases that are required for synthesis of omega-6 and omega-3 fatty acids. Therefore, expressing *Delta12 fatty acid desaturase* gene from spinach in pigs, which can effectively synthesize fatty acids, resulted in higher levels of linoleic acid (Saeki et al. 2004). In addition, researchers at the University of Missouri created pigs that produce their own omega-3 fatty acids, which are mainly found in fish oils and are beneficial to human health. These pigs express a humanized *Caenorhabditis elegans* gene, h*fat1,* which increased the omega-3 fatty acid composition compared to wild type controls (Lai et al. 2006).

### Hypoallergenic meat

In humans the gene responsible for terminal alpha 1,3 galacose residues on glycosylated proteins (Alpha 1,3 galactosyltransferase, *GGTA1*) is a non-functional pseudogene. Evidence suggests that certain tick bites in humans result in alpha-gal syndrome-a red meat allergy emanating from the alpha-gal residue on beef, pork, and lamb (Young et al. 2021). As a platform to develop swine for xenotransplantation, porcine *GGTA1* was disrupted by traditional homologous recombination in 2002 (Lai et al. 2002; Dai et al. 2002). In the homozygous null state, these pigs do not harbor terminal alpha 1,3 galactose (Phelps et al. 2003; Kolber-Simonds et al. 2004). Since red-meat allergy is due to an immune response to dietary alpha 1,3 galactose, this xenotransplantation platform would be a non-allergenic source of red meat. Recently, the U.S., Food and Drug Administration has approved the use of GalSafe® (Revivicor, LLC, Blacksburg, VA) pigs for human consumption (https://www.fda.gov/news-events/press-announcements/fda-approves-first-its-kind-intentional-genomic-alteration-line-domestic-pigs-both-human-food).

## Digestion improvement with genetically engineered pigs

Animal diets are the most economically important aspect of livestock production. Although catastrophic events such as floods or disease outbreaks may have a devastating impact in the short term, animal feed historically represents at least two thirds of total production costs. Multiple strategies can be used to increase overall feed efficiency. For example, increased growth rate can change the overall costs of maintenance and therefore will generally have a high impact on reduction of feed costs. However, in this section, the focus will be on genetic modifications that have a direct impact on digestion or gut content.

It has been known for decades that feedstuffs contain many macromolecules for which the production animal does not harbor a gene that encodes an enzyme for degradation. In fact, enzymes are often added to diets to increase overall nutrient availability. As with fed enzymes, manipulation of digestion via genetic engineering requires that the enzymes must be produced such that they are delivered to the digesta within the lumen of the gut, they survive the various pH conditions that may be encountered, they resist degradation by gut proteases, and that they function at physiological temperature. In addition to these qualities, genetically engineered digestive enzymes require that the proteins must tolerate the post-translational systems of the livestock host species. As an example, Hall et al., (1990) described an early attempt to produce a bacterial cellulase in cultured mammalian cells. Cellulose is the most abundant form of biomass on the planet, and livestock genome do possess genes for the enzymes to degrade this glucose polymer. From this effort, it was discovered that bacterial signal peptides may provide for adequate transport into the endoplasmic reticulum and that the host cells can glycosylate the protein. Further, this group produced transgenic mice with expression targeted to the exocrine pancreas (source of most endogenous digestive enzymes) and these mice did express a cellulolytic activity (Hall et al. 1993). However, the truncated form of the protein that was produced did not contain a cellulose binding domain. As such, the truncated bacterial cellulase could be detected on soluble laboratory substrates, but it was not active on substrates that are generally found in diets.

Adaptation of bacterial genes for mammalian secretion is possible. For example, to obtain enzymatic activity of the bacterial enzyme lysostaphin, the coding sequence had to be modified to prevent glycosylation in the mammalian secretory system (Kerr et al. 2001). To circumvent these types of issues, selection of digestive enzyme genes from other eukaryotes may be more productive. As an example, Lin et al. (2015) selected a cellulase gene from a fungus and appears to have succeeded in production of transgenic pigs that express an active cellulase. The authors clearly demonstrated cellulolytic activity on laboratory substrates. They also provide evidence of digestion of dietary fiber overall by analysis of feed and feces. However, it is not clear if the increased digestion of fiber had a direct effect on pig growth or if the effect was limited to gut microflora (Lin et al. 2015). In either case, the overall impact of this strategy seems to be very promising.

Although the overall utility of eukaryotes as a source of digestive enzyme genes is still unknown, it is clear that prokaryotic genes can be successfully employed. First in the mouse (Golovan et al. 2001a) and then in the pig (Golovan et al. 2001b) it was demonstrated that a bacterial phytase was fully functional as produced by the mammalian salivary gland. Phytic acid is the phosphorous storage molecule of plants and phytase is an enzyme that releases the phosphate groups. Vertebrates do not harbor a gene for phytase. In ruminants, bacteria provide multiple phytases. However, in swine and poultry, much of the organic phosphorous is lost to feces (environmental contaminant) while inorganic phosphorous must be added to diet to meet nutritional requirements (added cost). In these transgenic phytase pigs, the animal digests the phytic acid and does not require supplemental phosphorous (Meidinger et al. 2013). The distribution of phosphorous molecules in the waste has been characterized (Mao et al. 2008b). The transgene is stable over multiple generations (Forsberg et al. 2013). Importantly, the carcass and tissue nutrient distribution has been well characterized (Forsberg et al. 2014). However, since phytase is now inexpensively available as a feed additive and regulatory approval is slow and expensive, it appears that this technology may not have survived the bureaucracy.

The examples thus far involve digestive enzymes chosen to recover their products as nutrients. There are additional reasons to explore heterologous digestive enzymes. For example, most plant materials (and cereal grains in particular) contain non-starch polysaccharides (NPS). Livestock do not possess genes for the enzymatic activities required to digest NPS. Complete digestion of NPS would provide additional carbohydrate nutrients. However, the value of NPS digestion resides in physical changes to the digesta. One of the common classes of NPS is β-Glucans—these are the molecules that make some foods “slimy”. β-Glucans increase the viscosity of lumen contents, hold water in the gut, retain water soluble vitamins, and reduce nutrient absorption. Guan et al. have demonstrated first in the mouse (2013) and then in the pig (2017) that salivary production of a bacterial β-Glucanase can increase overall production efficiency. This group has further demonstrated that β-Glucanase can be delivered via the gut mucosa for similar benefits to digestion (Guan et al. 2013, 2017). A thorough comparison between the strategies has not yet been made.

This general strategy has been expanded to include expression of a fungal β-Xylanase. Xylan is a significant anti-nutritional factor for non-ruminants; β-Xylanase degrades xylan; and mammals do not harbor a gene that encodes β-Xylanase activity. Zhang et al. have shown that salivary delivery of β-Xylanase can increase feed efficiency (Zhang et al. 2018). However, additional studies are needed to characterize individual lines to better understand the full potential of this strategy. Similar strategies are being explored for Pectinases (Mo et al. 2019).

As noted above, feedstuffs may contain anti-nutritional factors for which transgenic strategies may provide solutions. However, feedstuffs may also contain toxic factors such as mycotoxins. To combat these fungal toxins, a bacterial gene that encodes and enzyme that detoxifies aflatoxin was expressed in the salivary gland of first the mouse (Guan et al. 2015) and then the pig (Lou et al. 2017). Unlike the experiments above that measured digestive parameters, these experiments required an examination of the impact of aflatoxin on the liver. Upon dietary challenge of the transgenic animals with aflatoxin, serum levels of total protein, albumin, globulin, alanine aminotransferase, and aspartate aminotransferase were measured. In addition, circulating levels of aflatoxin metabolites were also measured. The transgenic animals tolerated the Aflatoxin treatments better than non-transgenic control animals. A full description of these animals has not yet been published.

As with any other trait, production system would benefit from “stacking” traits onto the same genetic lines. Several groups have now begun to combine variations of the projects noted above so to create animals that have multiple, potentially beneficial genes. Zhang et al., characterized a variety of enzymes from multiple species to find a set of four genes that appear to express well in pig cells (Zhang et al. 2018). These four coding regions (two gluconases, a xylanase, and a phytase) were combined on a transposon to create a polycystronic transgene. All four enzymatic activities were observed in the transgenic animals produced. Using the same four gene cassette as above, Li et al. (2020) have adapted this general strategy to CRISPR technology. The main difference between the two strategies is that the transposon allows the researcher to survey many integration sites to find the best site amongst many (Li et al. 2020). The CRISPR strategy allows the researcher to place the cassette at a specific site that has been predetermined to be adequate. It is not clear if this strategy will allow for universally useful, multitransgenic animals or if specific sets of transgenes will be most efficient.

Other applications include methods to disseminate superior genetics into challenging environments (Park et al. 2017; Ciccarelli et al. 2020), altering the gut microbiome (Mo et al. 2021), digestion (Wang et al. 2020), reducing pollution (Forsberg et al. 2013), and to increase muscle growth (Li et al. 2020a, b). Benefits and application to the swine industry for each of these examples is different. In some cases, the economic benefit will be to the producer and in other cases a monetary justification may not be realized unless there is a financial disincentive to the disposal of, for example, phosphorous. Although some of the early experiments described in this section were completed in Europe or North America, it is clear that Asian researchers are now the most active in this field. As world demand for animal products grow, the overall efficiency of animal production must take advantage of every possible technology. Those countries that continue to hamper adoption of genetic engineering strategies will lose any leadership role that they have had in the past. The future of animal agriculture will be in the hands of those countries that recognize how to utilize genetic engineering in food animals.

## Somatic cell genome editing and gene therapy

Many of the same gene editing techniques used to improve aspects of pig agriculture have been employed in additional ways, including the development of pigs as biomedical models of human disease and their use as large animal models for testing the safety of gene editing reagents prior to their use in human clinical trials. Indeed, an essential component for developing human somatic cell gene editing technologies is the establishment of efficient and safe delivery reagents. Toward this goal, the NIH has recently established the Somatic Cell Genome Editing (SCGE) program (https://scge.mcw.edu/). For gene editing technologies to translate to clinical applications, large animal models (pigs and non-human primates) are an essential part of the SCGE program. The pig is particularly important in this effort. For many gene therapy-based efforts, scale-up is a significant issue. Unlike mice and most other models, pigs can be produced to span the entire human spectrum for size, weight, or blood volume at the level of the organism or at the level of the specific organ. In addition, the pig is intermediate between mice and primates in regard to immune responses and inflammation (Dawson et al. 2017, 2013; Starbaek et al. 2018). Consequently, pigs are becoming more widely used as the large animal component of preclinical trials (Badimon et al. 2019). With more gene edited (GE) pigs becoming available, swine have the potential to be a powerful tool for drug and device development (Boettcher et al. 2021; Cozzi et al. 2016; Donaldson et al. 2021; Hering and O'Connell 2016). Pigs are often preferable to dogs or primates for pharmacological reasons (Schook et al. 2015; Svendsen 2006). In Japan, pig use has surpassed dog use in clinical research (Kobayashi et al. 2012; Tanaka and Kobayashi 2006). Not expectedly, pig models are important for NIH’s Clinical and Translational Science Award initiatives (CTSA) and FDA’s Critical Path Initiative.​ Finally, the pig is the only large animal model for which genetic engineering technologies are robust. Although the strategies often differ, any genome modification that can be done in mice, can also be done in the pigs.

The NIH SCGE program is a cross-disciplinary, multi-center consortium for accelerating the use of genome editing technologies into clinical applications.

The Large Animal Testing Centers (LATC) of the SCGE are tasked to evaluate gene editing (GE) reagents and delivery methods created by other labs in the SCGE. To do this work, the swine LATC employs porcine cells, cell lines and live animals, including wildtype and swine models (transgenic animals possessing genes encoding for fluorescent proteins or other reporters, such as sodium-iodide symporter). The primary purpose of the swine LATC is to use these cells/animals to test genome editing reagents that have been proven effective in the mouse, ultimately in preparation for translating the delivery and editing technologies to the human clinic. In short, substantial investments in funding and effort are being devoted toward expanding the use of swine as models for human disease and as important animals for the evaluation of the safety of gene editing reagents prior to their use in humans.

## Understanding embryo development by using genome editing systems

Genome editing technology has furnished a novel opportunity to elucidate mechanistic events of embryogenesis. Interfering with the level of endogenous target genes in embryos is a key to understand mechanism of embryo development. Conventionally, technologies such as siRNA or morpholinos have been used to disrupt target genes (Lee et al. 2012, 2014; Huang et al. 2015). However, because embryos go through rapid cell divisions, it is difficult to effectively inactivate target genes in all embryonic blastomeres by using these approaches. Knock out embryos, on the other hand, present a more homogeneous population of cells. Unfortunately, obtaining knock out embryos generally would require the production of knock out animals, which is a prolonged process in livestock species. For instance, considering days to reach puberty and gestation period (114 days), a single round of breeding is typically over a year in pigs. Development of genome editing systems such as CRISPR/Cas9 system offers practical ways to study early embryo development because of their ability to introduce targeted modifications at a high efficiency and during embryogenesis. Specifically, the use of genome editing systems now allow us to produce knock out embryos without having to establish knock out pigs or a breeding program.

Pig conceptuses go through dramatic morphological changes during early development. Spherical conceptuses transform into a tubular and then filamentous form prior to attachment to the uterine surface (Geisert et al. 2015). The elongated conceptuses secrete various factors during attachment to the uterine surface epithelium that are essential for continued development and survival. Previous studies suggest that conceptus production of interleukin 1 beta 2 (IL1B2), estrogen, prostaglandins, and interferons are key signaling molecules for the development, attachment, immune regulation and establishment of pregnancy. However, dissecting the molecular pathways has been troublesome due to the lack of tools required to disrupt each pathway. Application of the CRISPR/Cas9 system allows one to disrupt target molecular pathways or genes, thus clarifying the role of conceptus factors for the establishment and maternal recognition of pregnancy. For instance, targeted disruption of *IL1B2* in somatic cells followed by SCNT produced embryos lacking functional IL1B2 and the conceptuses derived from the embryos failed to elongate in vivo, validating the importance of IL1B2 for conceptus survival and development (Whyte et al. 2018). A similar approach was used to examine the involvement of conceptus production and secretion of estrogen. By using the CRISPR/Cas9 system, aromatase, an enzyme responsible for the synthesis of estrogen, was inactivated in somatic cells, and embryos were produced through SCNT and transferred into surrogates (Meyer et al. 2019). Conceptuses developed and elongated in the uterus; however, the lack of estrogen synthesis did not prevent maintenance of the corpora lutea (maternal recognition of pregnancy) and formation of the placenta (Meyer et al. 2019), suggesting that additional conceptus factor(s) besides estrogen can extend and maintain corpus luteum function. However, all recipient gilts aborted between Day 25 and 30 of gestation. In addition, the CRISPR/Cas9 knock out of conceptus interferon gamma production causes a hyperinflammatory response within the uterus which resulted in conceptus fragmentation (Johns et al. 2021). The approaches have been successfully used to understand conceptus interaction(s) with the maternal uterus, expanding our knowledge on embryo development, establishment of pregnancy and survival. CRISPR/Cas9 system has facilitated the research as inactivation of both alleles, which considered intractable in pre-genome editing era, can now be effectively performed using the system.

As aforementioned, development of CRISPR/Cas9 system permit targeted modifications during early embryogenesis. Specifically, recent studies utilized CRISPR/Cas9 system to introduce targeted modifications during embryogenesis, therefore, generating knock out embryos without having to incorporate SCNT and efficiency of the targeting could reach as high as 100% (Whitworth et al. 2014; Lei et al. 2016). A recent study incorporated this approach to study the effect epigenetic modulators on the lineage specification in developing pig blastocysts. CRISPR/Cas9 was used to disrupt the tet methylcytosine dioxygenase 1 (*TET1*) gene, known to modulate the level of DNA methylation in developing embryos and the level of DNA methylation and expression of pluripotency related genes were explored in day 7 pig blastocysts (Uh et al. 2020). The CRISPR/Cas9 system induced targeted disruption of *TET1* at 100% efficiency and embryos lacking functional TET1 presented abnormal level of DNA methylation and transcript abundance of pluripotency genes in blastocysts, clarifying the role of TET1 in establishing lineage-specific differentiation in developing embryos. Since the *TET1* knock out embryos were produced without having to establish a herd of knock out pigs, it significantly reduced the time and effort required to obtain the embryos and study them. Similar approaches have been used in other species; targeted disruption of POU class 5 homeobox 1 (*POU5F1*) and Nanog homeobox (*NANOG*) by using CRISPR/Cas9 system illustrated their role on the lineage-specification of embryos (Ortega et al. 2020; Daigneault et al. 2018).

As demonstrated above, the development of genome editing systems provides an unprecedented opportunity to study detailed mechanistic actions of embryo development in livestock. The basic knowledge obtained from these studies will expand our overall understanding of animal models and be utilized to secure productivity of livestock species. Technical innovations to genome editing technologies will reduce any side effects associated with the technology further improve their use to elucidate biological events.

## Conclusions

Gene editing is explored across the livestock industry to improve animal welfare and increasing efficiency. Livestock genetic engineers quickly adapted gene editing into precision breeding protocols to increase the rate of change and make vast improvements. Pigs were the first livestock species that were successfully created to make the first disease resistant animals (Whitworth et al. 2016, 2018). This was flowed by creating pigs via precision breeding to improve production traits (Telugu et al. 2017), to lessen the impact on the environment (Forsberg et al. 2013), improve digestion (Guan et al. 2017) as well as to understand reproductive performance (Johns et al. 2021).

## Data Availability

All published references are available on Pubmed.

## Abbreviations

ANPEP: Amino peptidase N
ASFV: African Swine Fever Virus
*ATNXR1*: Anthrax toxin receptor 1
BE: Base editor
Cas: Clustered regularly interspaced palindromic repeats associated protein
CD163: Cluster of differentiation 163
CRISPR: Clustered regularly interspaced palindromic repeats
DSB: Double stranded breaks
ESC: Embryonic stem cell
*GGTA1*: Alpha 1,3 galactosyltransferase
hLZ: Human lysozyme
*hFAT1*: Fatty acid desaturase
IL1B2: Interleukin 1 beta 2
INDEL: Insertions or deletions
iPS: Induced pluripotent stem
LATC: Large Animal Testing Centers
NANOG: NANOG homeobox
NHEJ: Non-homologous end joining
NPS1: Non-starch polysaccharides
PAM: Protospacer adjacent motif
PAMS: Pulmonary alveolar macrophages
PDCoV: Porcine deltacoronavirus
PEDV: Porcine epidemic diarrhea virus
pegRNA: Prime editing guide RNA
POU5F1: POU class 5 homeobox 1
PRRSV: Porcine reproductive and respiratory syndrome virus
*pRSAD2*: Porcine radical SAM domain-containing 2
RELA: RELA proto-oncogene, NF-kB subunit
RT: Reverse transcriptase
SCGE: Somatic Cell Genome Editing
SCNT: Somatic cell nuclear transfer
sgRNA: Single guide RNA
SIGLEC1: Sialic acid binding Ig like lectin 1
SVA: Senecavirus A
TALEN: Transcription activator: effector nuclease
TET1: The tet methylcytosine dioxygenase 1
TGEV: Transmissible gastroenteritis virus
ZFN: Zinc finger nuclease
*TMPRSS2*: Transmembrane serine protease 2
UCP1: Uncoupling protein 1
